# Thoracic endovascular aortic repair-first hybrid repair without thoracotomy for type V thoracoabdominal aortic aneurysm

**DOI:** 10.1016/j.jvscit.2026.102523

**Published:** 2026-09-19

**Authors:** Reo Fukunaga, Takao Ohki, Kota Shukuzawa, Hirotsugu Ozawa, Kentaro Kasa, Ryo Nishide, Daisuke Yamagishi

**Affiliations:** a The Jikei University School of Medicine, Tokyo, Japan; b Division of Vascular Surgery, Department of Surgery, the Jikei University School of Medicine, Tokyo, Japan

**Keywords:** Thoracoabdominal aortic aneurysm, Type Ⅴ, Hybrid repair, TEVAR-first, Thoracotomy-free surgery

## Abstract

We report a case of type V thoracoabdominal aortic aneurysm treated using two-stage hybrid repair. A 47-year-old man had a history of left nephrectomy for hydronephrosis in early childhood, Takayasu arteritis, and total arch replacement for ascending aortic aneurysm. He first underwent zone 4 thoracic endovascular aortic repair, followed by open surgery for the thoracoabdominal aortic aneurysm without thoracotomy. The previously deployed stent graft served as the proximal clamping site, enabling the proximal anastomosis to be performed below the diaphragm. This hybrid approach enabled surgical repair without thoracoabdominal incision or diaphragmotomy, which are known to cause short- and long-term side effects.

Open surgical repair of thoracoabdominal aortic aneurysm (TAAA) remains one of the most complex and technically demanding procedures in vascular surgery. Even in high-volume specialized centers, perioperative mortality rates of 5% to 15% have been reported.^1^, ^2^, ^3^ Major complications include respiratory failure, spinal cord ischemia, and renal failure, among which respiratory failure is particularly prevalent, with reported incidence rates of up to 60%.^1^, ^2^, ^3^ Given these substantial risks, endovascular repair has increasingly emerged as an alternative treatment strategy for TAAAs in recent years. However, the indications for endovascular repair and its clinical outcomes remain limited in the current literature.^4^

Type V TAAA, which involves the lower descending thoracic aorta and visceral segment while sparing the infrarenal aorta,^5^ provides a unique opportunity for limited but strategic intervention. Hybrid repair, such as debranching thoracic endovascular aortic repair (TEVAR), has emerged as a promising option, especially when an adequate proximal landing zone is available and the anatomy allows extra-anatomical or visceral-preserving reconstruction.^6^, ^7^, ^8^, ^9^, ^10^, ^11^, ^12^

Herein, we report a case of a patient with type V TAAA who underwent successful TEVAR-first hybrid repair. This strategy avoided thoracoabdominal incision and preserved diaphragmatic integrity, thereby contributing to early recovery and the avoidance of pulmonary complications as well as other long-term consequences associated with diaphragmotomy.

## Case report

Written consent was obtained from the patient for the publication of the details and images of this case.

A 47-year-old man was referred to our institution for the treatment of TAAA. He had a significant medical history of Takayasu arteritis and had undergone multiple prior cardiovascular surgeries, including aortic valve replacement for aortic valve stenosis and coronary artery bypass grafting at the age of 34, as well as total aortic arch replacement for an aortic aneurysm at the age of 42. He also had a history of left nephrectomy for hydronephrosis at the age of 3, renovascular hypertension treated with right renal artery stenting at the age of 30, chronic kidney disease (estimated glomerular filtration rate, 48 mL/min/1.73 m^2^), and carotid artery stenosis under surveillance. His medications included prednisolone, methotrexate, warfarin, aspirin, nifedipine, and carvedilol. He had no respiratory symptoms, and pulmonary function was preserved.

Computed tomography angiography (CTA) revealed a 60-mm fusiform aneurysm of the thoracoabdominal aorta extending from the level of the 11th thoracic vertebra (T11) to the level of the right renal artery (Fig 1). The descending aorta proximal to the TAAA showed a relatively constricted segment of approximately 18 mm in length, above which a 42-mm descending thoracic aortic aneurysm was present. The aneurysm involved the origins of the celiac trunk and superior mesenteric artery.

**Fig 1:**
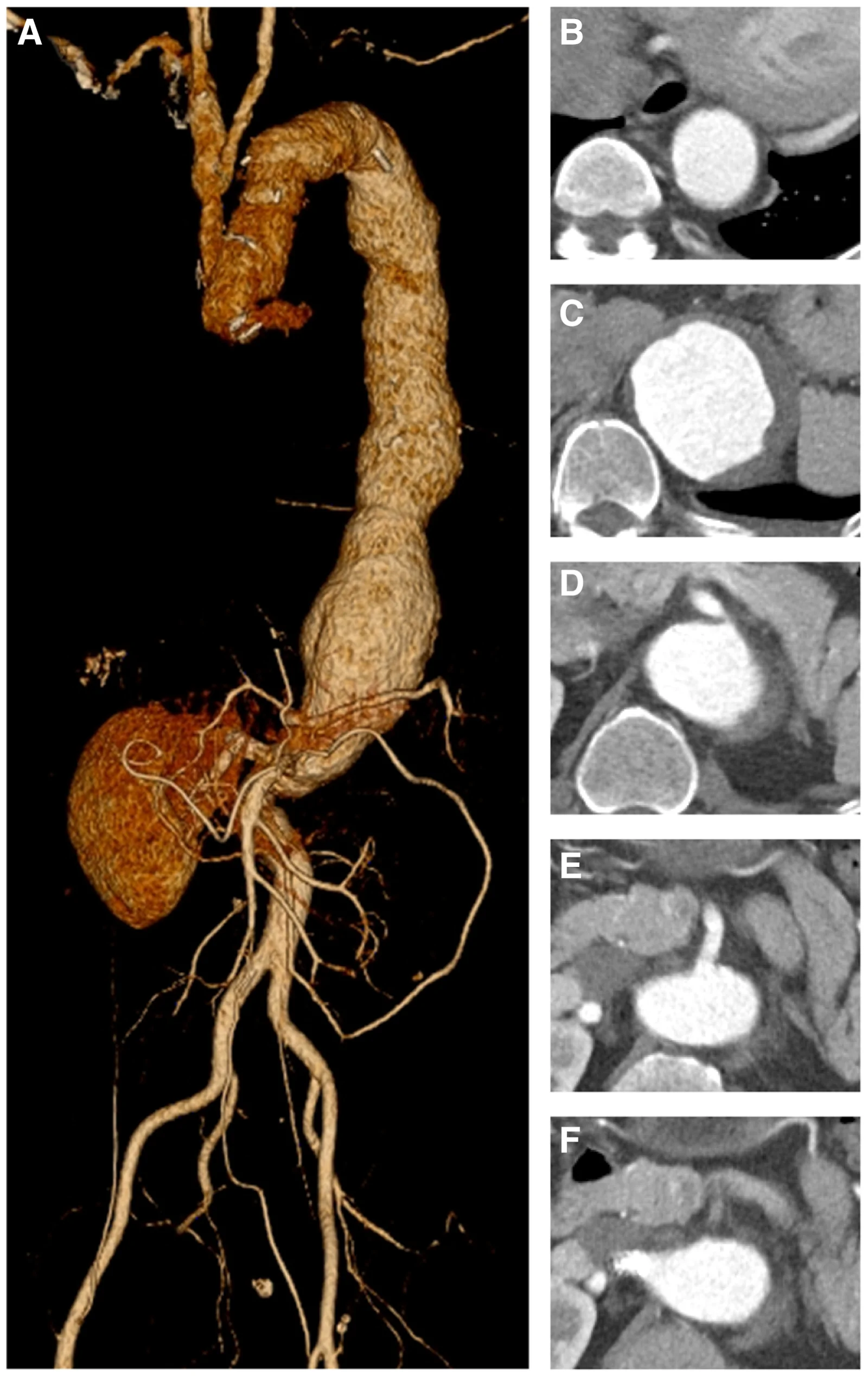
Preoperative computed tomography angiography (CTA). **A,** Three-dimensional reconstruction of the thoracoabdominal aortic aneurysm. **B,** Axial image of the proximal neck at the level of the 11th thoracic vertebra (T11) (42 mm). **C,** Axial image of the aneurysm (maximum diameter, 60 mm). **D,** Axial image of the aorta at the level of the celiac trunk. **E,** Axial image of the aorta at the level of the superior mesenteric artery. **F,** Axial image of the aorta at the level of the right renal artery.

Given the history of previous thoracic surgeries and the need to minimize surgical invasiveness, we planned a two-stage hybrid repair consisting of initial TEVAR followed by open surgical replacement without thoracotomy and diaphragmotomy. This staged strategy was intended to reduce the risks of pulmonary complications, spinal cord ischemia, and postoperative pain, while preserving a virgin thoracic field for potential future intervention for residual thoracic aortic dilation.

### Operative procedure

The patient initially underwent TEVAR via right femoral artery access under general anesthesia. A 40-mm × 100-mm TAG conformable thoracic stent graft (W.L. Gore and Associates) was deployed in the descending thoracic aorta at the level of T10 (Fig 2, *A* and *B*), using the constricted segment just above the aneurysm as the proximal sealing zone. The procedure was completed without balloon dilation or additional fixation, as the device was intended to serve as an intraoperative clamping site and proximal site for anastomosis during the second-stage open surgery. A three-dimensional image of postoperative CTA is shown in Fig 2, *C*.

**Fig 2:**
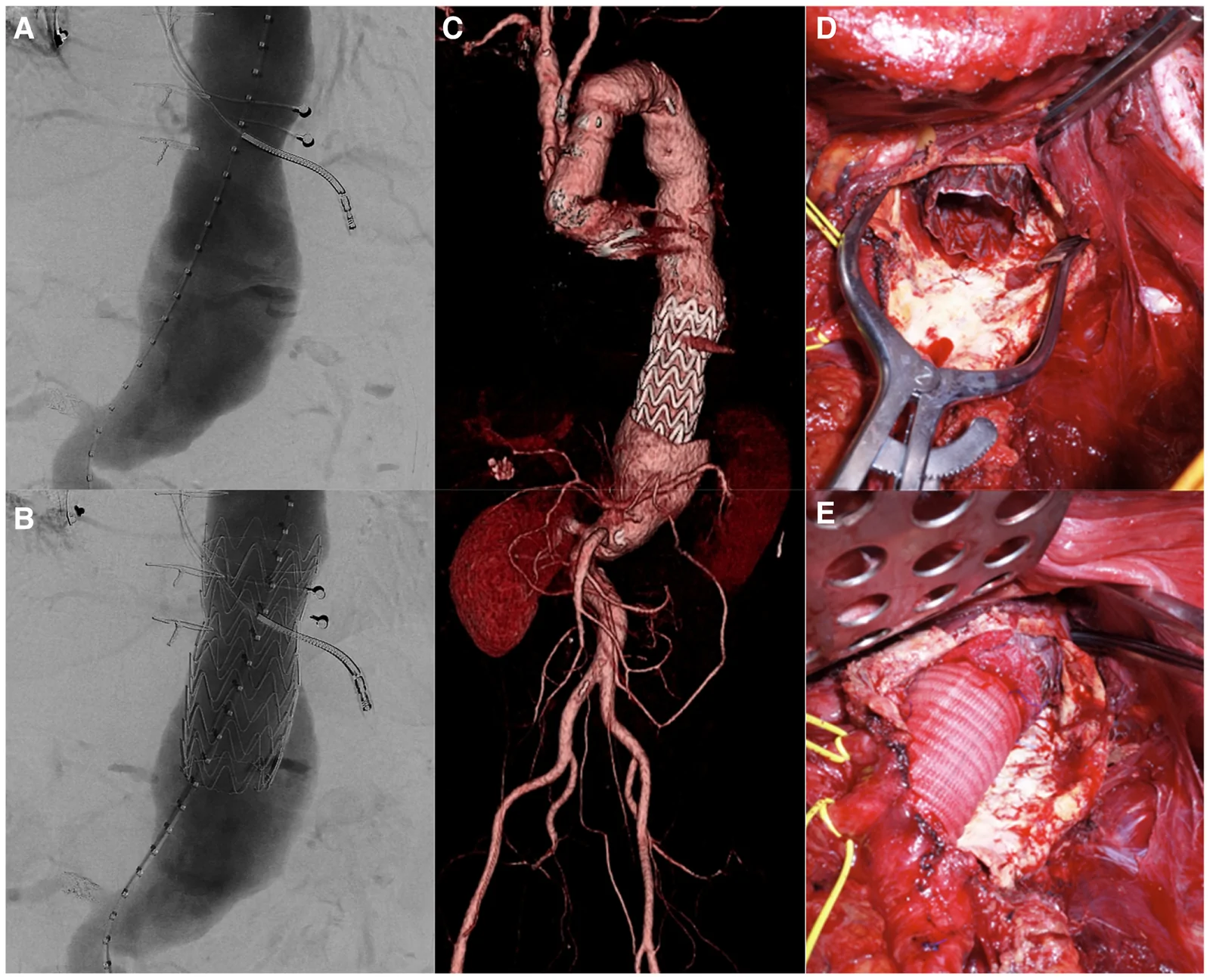
Thoracic endovascular aortic repair (TEVAR)-first hybrid repair. **A,** Preoperative angiogram during the initial TEVAR. **B,** Completion angiogram after the initial TEVAR. **C,** Three-dimensional reconstruction of computed tomography angiography (CTA) after the initial TEVAR. **D,** Cross-clamping performed directly over the stent graft with the aneurysm sac opened. **E,** Proximal anastomosis between the stent graft and a Dacron graft, with visceral branch reconstruction using with a beveled distal anastomosis.

Eleven days after the initial TEVAR, the patient underwent the second-stage open surgery. Through a spiral skin incision at the left ninth intercostal space and using a retroperitoneal approach, the thoracoabdominal aorta was clearly exposed while preserving diaphragmatic integrity and avoiding thoracotomy. Cross-clamping was performed directly over the stent graft at the constricted segment, thereby eliminating the risk of dissection or aortic wall damage associated with clamping the native diseased aorta (Fig 2, *D*).

The aneurysm sac was opened, and the proximal suture line was constructed without incorporating the native aortic wall. After felt reinforcement of the stump, the distal end of the previously placed stent graft was anastomosed to a 30-mm J Graft (Japan Lifeline Co, Ltd). A beveled distal anastomosis was constructed to preserve the celiac trunk, superior mesenteric artery, and right renal artery (Fig 2, *E*). No distal perfusion system and no selective visceral or renal perfusion were used during the reconstruction. The proximal clamp time was 62 minutes, the visceral ischemia time was 77 minutes, and the lower limb ischemia time was 96 minutes.

### Postoperative course

The patient was extubated immediately after surgery and then transferred to the intensive care unit. The patient was managed according to our standardized spinal cord ischemia prevention protocol, including perioperative permissive hypertension. Prophylactic cerebrospinal fluid drainage and neuromonitoring were not performed, but cerebrospinal fluid drainage was planned to be promptly performed if paraplegia or paraparesis occurred. The postoperative course was stable without respiratory complications or spinal cord ischemia. Temporary hemodialysis was required for transient renal dysfunction, but renal function recovered, and the patient was discharged on postoperative day 20. Follow-up CTA 6 months after surgery confirmed complete exclusion of the aneurysm and patency of all visceral branches (Fig 3, *A-D*). The patient reported no postoperative wound pain, and the surgical scar healed well (Fig 3, *E*) with no decline in respiratory function.

**Fig 3:**
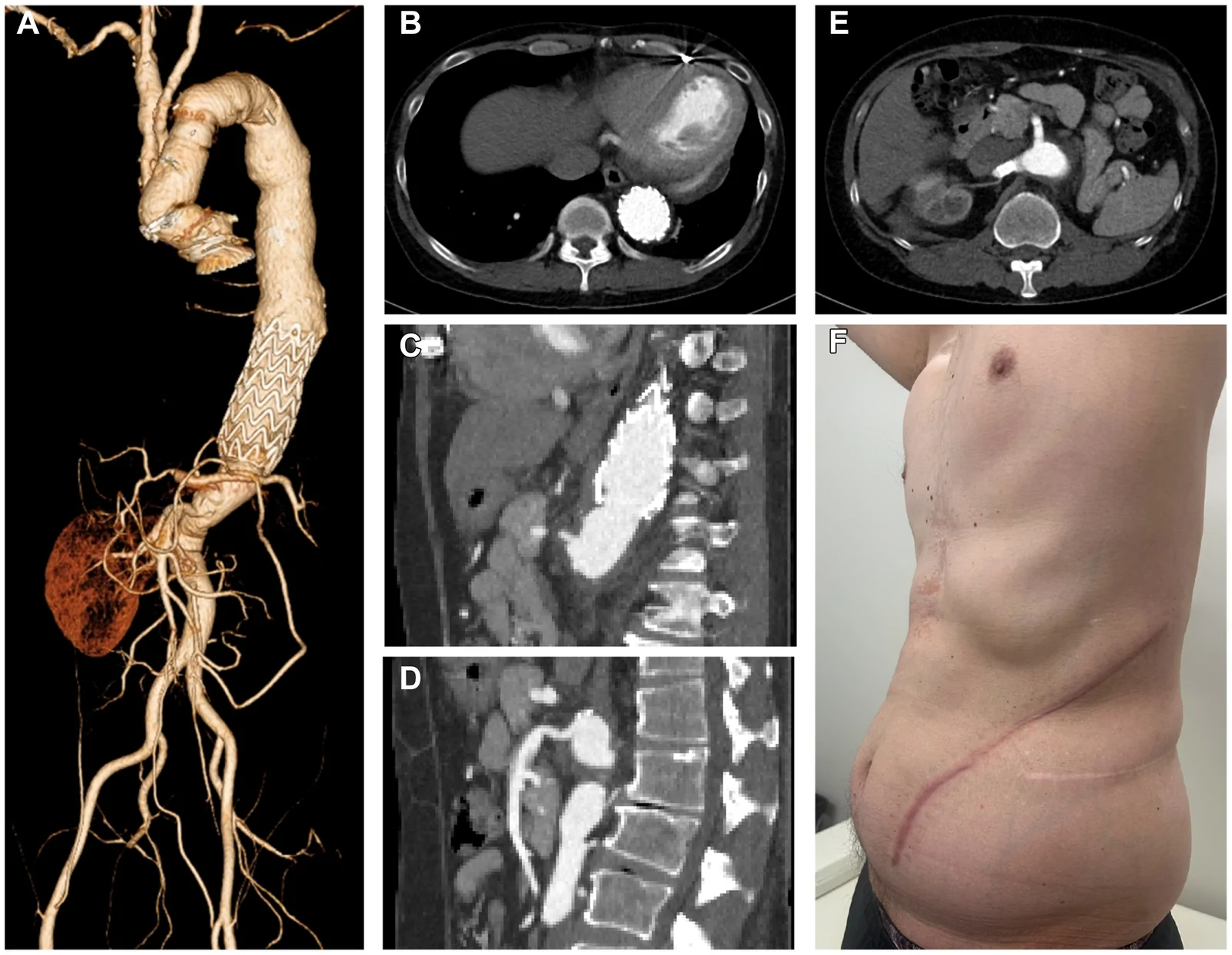
Postoperative computed tomography angiography (CTA) and surgical scar. **A,** Three-dimensional reconstruction of the thoracoabdominal aorta. **B,** Axial image of the aorta at the proximal neck. **C,** Sagittal image of the aorta at the level of the celiac trunk. **D,** Sagittal image of the aorta at the level of the superior mesenteric artery. **E,** Axial image of the aorta at the level of the superior mesenteric artery and right renal artery. **F,** Postoperative surgical scar.

## Discussion

This case demonstrates the feasibility of TEVAR-first hybrid repair for the treatment of type V TAAA. By performing initial TEVAR, the stent graft could be used as a secure proximal clamping and anastomotic site during the subsequent open procedure. This approach may contribute to reducing respiratory morbidity, spinal cord ischemia, and postoperative pain by avoiding thoracotomy and diaphragmotomy. In this particular case, without such a hybrid repair, aortic cross-clamping above the descending thoracic aortic aneurysm would have been required, thereby necessitating a thoracotomy. In addition, because the aorta proximal to the TEVAR sealing zone was aneurysmal, conventional open repair would likely have required more proximal clamping beyond the dilated segment and a more extensive graft replacement. This could have required sacrifice or reconstruction of additional intercostal arteries, thereby increasing procedural complexity from the standpoint of spinal cord protection. In contrast, the initial TEVAR enabled limited proximal control below the diaphragm while preserving the option of future proximal extension as part of a staged strategy.

Open surgical repair of TAAA remains associated with substantial perioperative morbidity and mortality, even in contemporary practice at high-volume referral centers.^1^, ^2^, ^3^ The extensive surgical exposure required for conventional open repair, including thoracotomy and diaphragmotomy, is a major contributor to postoperative respiratory failure, which continues to represent one of the most frequent and clinically significant complications. This issue is particularly relevant in patients with prior thoracic operations or underlying inflammatory aortic disease, in whom multiple prior surgeries further increase pulmonary morbidity and overall surgical risk. In contrast, endovascular repair has been shown to reduce the risk of such procedure-related complications, particularly by avoiding extensive thoracic exposure. In the context of TAAA, staged endovascular strategies have been reported to be effective in selected patients.^6^ However the application of endovascular repair to complex TAAA anatomy remains limited by anatomical constraints and insufficient long-term outcome data^13^ as well as the absence of approved branched stent grafts at the time when this patient underwent surgery.

In recent years, off-the-shelf multibranched endografts have emerged as an increasingly adopted alternative for TAAA repair, offering the advantage of immediate availability without the manufacturing delays inherent to patient-specific devices. Multiple series have demonstrated promising technical success rates across both elective and urgent presentations, including applications beyond the manufacturer's instructions for use.^14^, ^15^, ^16^, ^17^ However, these devices are not without limitations: anatomical feasibility is restricted by their standardized configurations and varies considerably depending on individual patient anatomy, as highlighted in a systematic review of currently available off-the-shelf platforms.^18^^,^^19^ The extensive aortic coverage required carries an increased risk of spinal cord ischemia compared with custom-made devices, and target vessel instability during follow-up, particularly at the renal artery level, remains a recognized concern. Furthermore, the extremely high cost of such devices is of concern. As such, although off-the-shelf multibranched endografts represent a valuable and expanding option, careful patient selection and institutional expertise remain essential. In the present case, however, multibranched endovascular devices were not reimbursed or clinically available for routine use in Japan at the time of treatment; therefore, this option could not be selected. Hybrid surgical strategies, such as the one described in this report, may continue to play an important complementary role when fully endovascular repair is anatomically unsuitable or clinically unavailable.

Type V TAAA represents a distinct anatomical configuration in which hybrid repair may be considered. Because the aneurysm involves the lower descending thoracic aorta and visceral segment while sparing the infrarenal aorta, extensive thoracoabdominal exposure can often be avoided if an adequate proximal landing zone is present. In such cases, hybrid repair, including debranching or staged TEVAR, offers the potential to limit surgical invasiveness while maintaining reliable visceral revascularization.^6^, ^7^, ^8^, ^9^, ^10^, ^11^, ^12^ Previous reports have demonstrated the feasibility of these strategies in selected patients, particularly when the proximal descending aorta is relatively preserved and suitable for endovascular fixation.^20^, ^21^, ^22^, ^23^

From a technical perspective, the key feature of the present strategy lies in the use of the previously deployed stent graft as a proximal clamping and a stable site for anastomosis. Cross-clamping directly over the stent graft provided reliable proximal control while avoiding manipulation of the native aorta, thereby reducing the risk of aortic injury. This hybrid approach enabled a controlled operative field and facilitated safe visceral revascularization while minimizing surgical invasiveness. Furthermore, under favorable anatomical conditions, this concept may have potential applicability to more extensive aneurysms, including Crawford type I or II TAAA, as part of a staged repair strategy.

Although conventional open repair should also be carefully considered in the present case, given the patient’s young age, Takayasu arteritis, and proximal descending thoracic aortic dilation, a relatively constricted segment of the descending thoracic aorta allowed limited proximal sealing for the initial TEVAR. The proximal sealing zone was ectatic and may not provide durable long-term sealing; therefore, this TEVAR-first approach should be regarded as part of a staged strategy rather than a definitive treatment for the entire thoracic aorta. Further proximal extension will be considered if progressive proximal aortic dilation or loss of seal is observed during follow-up.

This report describes a single case and focuses on a technical concept rather than broader clinical applicability. Accordingly, careful patient selection is essential, particularly with respect to anatomical suitability, the availability of an adequate proximal landing zone, and the risk of future degeneration of the residual native visceral aortic segment. Genetic testing for hereditary aortopathy could not be confirmed from the available medical records, and the possibility of an underlying genetic aortopathy should be considered during long-term follow-up. Nevertheless, this case contributes to the evolving landscape of TAAA interventions, and further studies are warranted to clarify the role of TEVAR-first hybrid repair in selected patients.

## Conclusions

This case demonstrates the feasibility of TEVAR-first hybrid repair of type V TAAA, in which initial TEVAR followed by open surgery enabled definitive surgical repair without thoracotomy or diaphragmotomy.

## Funding

None.

## Disclosures

None.
